# Correlation between ASA Grade with reversal of Hartmann’s procedure – a retrospective study

**DOI:** 10.25122/jml-2020-0158

**Published:** 2021

**Authors:** Muhaned Farah, Paolo Sorelli, Rajab Kerwat, Okatokundo Oke, Philip Ng

**Affiliations:** 1. Department of Colorectal Surgery, Lewisham and Greenwich Trust, London, United Kingdom

**Keywords:** Hartmann’s procedure, colorectal surgery, ASA grade, reversal of Hartmann’s rate

## Abstract

Hartmann’s procedure involves resecting the rectosigmoid colon, closure of the distal rectal stump, and forming an end colostomy for complicated left colon diverticulitis or malignancy. Recovery from the initial operation can, in a second stage, be followed by a reversal stage with the restoration of bowel continuity. This study aimed to assess the reversal rate and its correlation with demographic data, ASA grade, and length of hospital stay. All patients who underwent Hartmann’s emergency procedure from 2014 to 2018 at Lewisham and Greenwich hospital were enrolled in this retrospective study. Data was collected from the inpatient electronic files and NELA (UK National Laparotomy Audit). 118 patients were included in the study, with 57.6% females and a median age of patients of 69 years (range 35–91). Findings of the study indicate that the most common indications for Hartmann’s procedure were diverticular complications 60% (n=71) and benign perforated sigmoid or rectosigmoid cancer 16% (n=19). The average length of hospital stay was 24 days (range n=2 – 212 days). The reversal rate was 34.9% (41/118 cases). No significant difference was observed between gender and length of hospital stay in relation to the reversal rate while there was a significant correlation between age and ASA grade in relation to reversal rate; the calculated P values were recorded as (<0.000) and (<0.009) respectively. Our results show that the highest reversal rate was observed in younger and fitter (I–II) ASA grade patients. The most common medical complication from reversal of Hartmann’s procedure was an anastomotic leak (n=6, 16.7%). Reversal rate of Hartmann’s procedure was 34.9%. The average timeframe for reversal was within 18–20 months. There was a significant correlation between age and ASA grade in relation to reversal rate.

## Introduction

In the early twenties of the past century, the French surgeon Henri Albert Hartmann introduced and described a surgical procedure for obstructive rectosigmoid cancer in 1921, with postoperative sequel superordinate for other surgical techniques. Hartmann’s procedure is defined as a surgical procedure that mainly includes rectosigmoid resection and closure of rectal stump plus establishment of an end colostomy [1]. It is used for many indications, where other surgical approaches are considered less safe [2, 3]. Moreover, in acute conditions such as obstructed left colon as a result of malignancy, left colonic perforations, diverticular disease; Hartmann’s procedure is presently the operation of choice for these conditions as it significantly reduces the chance of anastomotic leakages associated with the classic primary anastomosis [4, 5].

American Society of Anesthesiologists Physical Status (ASA PS) classification was first implemented in clinical practice more than seventy years ago to classify patients according to tolerance and fitness for specific surgical procedures. The ASA class is a subjective evaluation of a patient’s overall health, and it only measures the amount of physiological allowance that a patient has at the time they are evaluated for a surgical procedure. Also, it should not be used as the only indicator of an operative risk to the patient [6]. ASA score consists of five classes (I to V) as follows: in class I, the patient is completely fit for surgical operation, class II patient has only mild systemic disease, class III patient has severe systemic disease that is not disabling, class IV patient has severe systemic disease that confers impending threat to life, class V a severe morbid condition that the patient is not anticipated to live 24 hours with or without surgery [7]. Reversal of Hartmann’s procedure and restoration of colonic continuity was associated with relatively high morbidity and mortality rates [8, 9]. Reversal of Hartmann’s procedure could be done by either open surgery approach or laparoscopic approach [10]. With the increasing practice of laparoscopic colonic surgery, a global reduction in operative morbidity was noted [11]. Most morbidity and mortality cases are due to complications such as an anastomotic leak, wound dehiscence, and failure of colostomy reversal [12]. Other reported complications include the formation of intra-abdominal abscess, wound infection, an ileus, and incisional hernia [13–15]. Factors associated with Hartmann’s procedure reversal outcomes are ASA class, patient age, and tumor staging [16]. The ideal time for Hartman procedure reversal remains controversial. Some surgeons suggest that reversal should be done by 15 weeks to lower the risk of postoperative complications [17], while others advocate reversal after 6 months, allowing adhesions to soften, which will reduce operative difficulty [18].

Our study aims to identify the factors that affect the reversal rate, mainly the age, gender and ASA grade, and length of hospital stay [16]. In addition, we are interested in studying the impact of the initial pathology diagnosis on the reversal rate, as our sample comprises a heterogeneous population of patients, including diverticular diseases and malignant diseases.

## Material and Methods

This is a retrospective observational study. All patients who underwent emergency Hartmann’s procedure between 2014 and 2018 at Lewisham and Greenwich hospitals were identified and included. We excluded patients who have their Hartmann’s procedure on an elective basis. The cases were retrieved from a computerized medical records database and NELA (UK National Laparotomy Audit) bowel database. Analysis was completed by examining the inpatient files in selected patients where appropriate. Collected data included the following parameters: demographics, indications for Hartmann’s procedure, duration of the surgery, hospital stay, ASA grade, and reversal rate. In addition, the type of surgery (open or laparoscopic) covering ileostomy and postoperative complications was collected for patients who underwent a reversal. All the reversal procedures were performed by qualified colorectal surgeons, while the original Hartmann’s procedure was performed by a mixture of emergency surgeons, general surgeons, and colorectal surgeons. Data analysis was performed using the SPSS software. A chi-squared test was used to assess categorical variables. A p-value of less than <0.05 was considered to be statistically significant.

## Results

**Figure 1.**
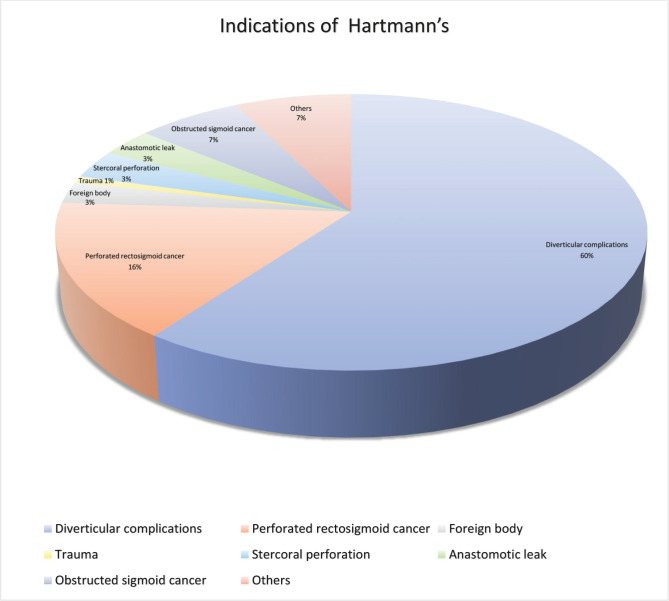
Hartmann’s procedure indications.

**Figure 2.**
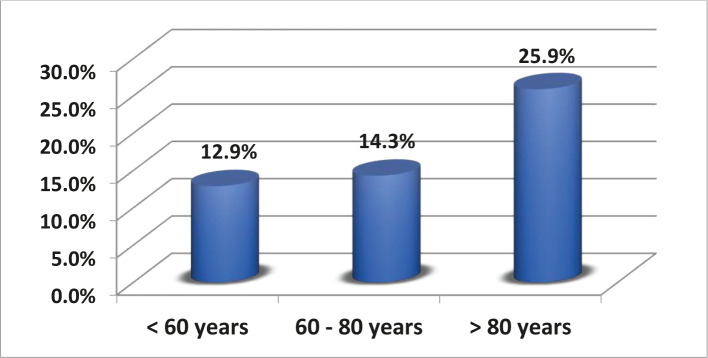
30-day mortality in different age groups.

A total of 118 patients were identified during the study period from 2014 to 2018 at Lewisham and Greenwich hospital. 68 (57.6%) of the 118 patients were female. The median age was 69 years (range from 35 to 91). A substantial proportion of patients were ASA grade III–V (n=72, 59.4%), as summarized in Table 1. The most common indications for Hartmann’s procedure (Figure 1) were diverticular complications (n=71, 60%), benign perforated sigmoid (stercoral and traumatic) or rectosigmoid cancer (n=19, 16%), other indications such as obstructed sigmoid cancer (n=8, 7%). The mean duration of surgery was 2h. 24 min (range 53–332 min), and the average length of hospital stay following was 24 days (range 2–212 days). 30-day mortality after the index Hartmann’s procedure was 16.9% (20/118 cases) and was predictably higher in those aged above 80 years when compared with the other age groups (Figure 2). 

**Table 1.** Patient’s demographics, mortality, and reversal rates.

		**Frequency**	**Percent**
**Gender**	Male	49	41.5%
	Female	68	57.6%
	Missing	1	0.8%
**Age (years)**	<60	29	24.5%
	60–80	62	52.5%
	>80	27	22.8%
**ASA grade**	I–II	48	40.6%
	III–V	70	59.4%
**Mortality rate**	Yes	20	16.9%
	No	98	83.1%
**Reversal rate**	Reversed	41	34.7%
	Not reversed	77	65.2%

### Reversal rate of Hartmann’s procedure

**Figure 3.**
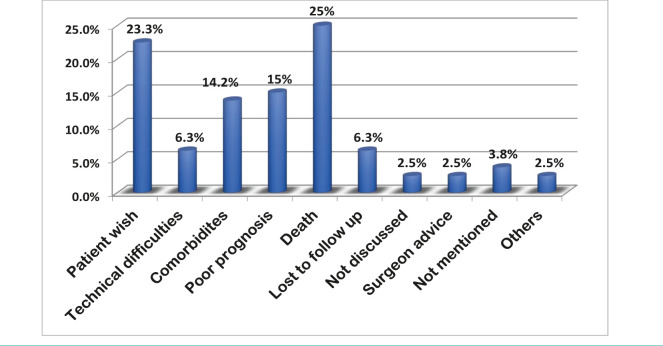
Causes of no reversal of Hartmann’s procedure.

Sigmoidoscopy to assess the rectal stump length and colonoscopy through the stoma to rule out residual proximal pathology were routinely performed prior to reversal. The reversal rate of Hartmann’s procedure was 34.7% (41/118 cases). Seventy-seven patients (65%) did not proceed to reversal (Table 1). The reason for patients not being reversed were patient’s death (n=20, 25.9%), patient wish (n=18, 23.3%), recurrent malignancy (n=12, 15.5%) or unfit for surgery due to extensive comorbidities (n=11, 14.2%) (Figure 3).

Forty-one patients underwent successful reversal (34.9%). Defunctioning ileostomy was used in 10 patients (24%), an anastomotic leak occurred in 6 patients (14.6%). The operative time mean was 3 hrs.15 min, and the average length of hospital stay following reversal was 11 days (range 2–159 days). 

### Correlation between gender, age, ASA grade, length of hospital stay, and reversal rate

The Chi-square test was used to determine the significance of differences between the reversal rate of Hartmann’s procedure and the demographic data of the patients, ASA grade, and length of hospital stay (Table 2). No significant difference was observed between gender and length of hospital stay in relation to the reversal rate, as the calculated p-values for these parameters were recorded as <0.737 and <0.840, respectively. On the contrary, there was a significant correlation between age and ASA grade in relation to reversal rate (p-value <0.001 and <0.009, respectively). Our results showed that the highest reversal rate was observed in younger and (I–II) ASA grade patients, while the lowest reversal rate was observed in patients aged above 80 years and patients with (III–V) ASA grade.

**Table 2.** Correlation between demographics, ASA grade, length of hospital stay, and reversal rate.

**Characteristic**	**Reversal rate**		**P-value**
	**Yes**	**No**	
	**N (%)**	**N (%)**	
**Gender**			
**Male**	19 (38.8)	30 (61.2)	<0.737
**Female**	22 (32.3)	46 (67.6)	
**Age (Years)**			
**<60**	19 (61.3)	12 (38.7)	<0.001
**60–80**	21 (33)	42 (66.7)	
**>80**	1 (3.7)	26 (96.3)	
**ASA grade**			
**I–II**	24 (49)	25 (51)	<0.009
**III–V**	17 (23.6)	55 (76.4)	
**Length of hospital stay (days)**			
**<20**	25 (34.2)	48 (65.8)	<0.840
**20–40**	9 (33.3)	18 (66.7)	
**41–60**	3 (25)	9 (75)	
**>60**	4 (44.4)	5 (55.6)	

## Discussion

This study involved the selection of 118 consecutive patients who underwent Hartmann’s procedure to measure the reversal rate and mortality rate and assess the correlation between demographic data, ASA grade, length of hospital stay, and the reversal rate of Hartmann’s procedure. The most common indication for Hartmann’s procedure in our study was diverticular complications (60%), similar to the studies conducted by Hallam *et al.* [19] and Christou *et al.* [20], while the most common indication for Hartmann’s procedure in the study conducted by Zarnescu *et al.* [21] was colorectal cancer.

In this study, the reversal rate of Hartmann’s procedure was assessed. 34.9% of the patients underwent a successful reversal, which is higher to previously reported data by Christou *et al.* [20], where only seventy patients (29.2%) underwent a reversal. A higher percentage was reported in the study conducted by Hallam *et al.* [19], who reported that the reversal of Hartmann’s rate was 47%. A group of patients wished not to be reversed; their decline was based on their hospital experience. This is the group with the longest hospital stay after Hartmann’s procedure; most of them have been to ITU, which caused stressful experiences complicated by post-traumatic stress syndrome. This is an area of interest to study the factors that influence a patient’s decision to reversal. 

Thirty-day mortality following Hartmann’s procedure was 16.9%; it was higher in those aged above 80 years. This was in accordance with previously reported results by Zarnescu *et al.* (16.1%) [21]. It would be reasonable to expect that advances in surgical, critical care, and antimicrobial treatment would have resulted in improvements in these dreadful percentages. In our study, half of the reversals were approached laparoscopically. Due to technical difficulties and dense adhesions, one-third of those patients were converted to open surgery. A systematic review by Siddiqui *et al.* observed a low rate of laparoscopic surgery for a colostomy reversal. However, good results have been published with its use, and a laparoscopic reversal is comparable or superior to open reversal [22]. Post reversal complications occurred in 6 patients (14.6%). Anastomotic leak was the most common surgical complication (14.6%), while the most common complication reported by Zarnescu *et al.* [21] was diarrhea (n=4, 7.2%). Most of the leaks were minor and were managed with intravenous antibiotics and percutaneous drainage; only one patient had a major leak which required laparotomy, taking down the anastomosis, and re-stoma formation. 

Our results revealed that there was no significant difference between gender and reversal rate (p<0.737), while a significant relation was observed between age (p<0.001) and ASA grade (p<0.009) in relation to reversal rate, the highest reversal rate was observed in younger and (I–II) ASA grade patients. In addition, there was a higher rate of reversal in benign conditions than malignant conditions. These results were in accordance with previous results reported by Hallam *et al.* [19], where there was an increased likelihood of reversal if the patients were younger (p<0.001) and ASA grade less than or equal to 2 (p<0.0001); gender was not significantly associated with a reversal.

There are several other surgical trends to approach the management of diverticular complications or obstructed rectosigmoid tumors, such as colonic stent insertion and primary anastomosis with or without a stoma. The colonic stent can convert the emergency surgery to a semi-elective surgery which allows more time for patient optimization and a procedure performed by a colorectal surgeon. Primary anastomosis with or without diverting ileostomy is another option that can save the patient from undergoing another major and morbid surgery. This latter approach was not adopted in our current study as most of Hartmann’s procedures were performed by emergency consultants who are not specialized in colorectal surgery and only occasionally perform colon resections. On the other hand, colorectal surgeons performed all reversal operations, who conducted primary anastomosis and diverting ileostomy in one-quarter of the reversed patients. Moving towards centralizing services with 24 hours access to colorectal surgeons may reduce the number of Hartmann’s procedures performed overall. In addition, providing up-to-date information on the management of those common problems in the surgical department is crucial.

One limitation of our study is the retrospective data collection from a dual centers group, involving many surgeons not necessarily specialized in colorectal surgery. Second, our patient group was rather heterogeneous. However, this variety in the patient mix was a caveat to assess possible risk factors for non-reversal. The value of our study is that it demonstrates that Hartmann’s procedure is still a very prevalent surgery that carries high mortality and morbidity, but it can still save lives in high-risk comorbid patients, and by implementing new evolving surgical options, we can improve the quality of patient care.

## Conclusion

In conclusion, Hartmann’s procedure is still a commonly performed emergency colorectal operation. Reversal rate of Hartmann’s procedure was 34.9%. The predicted mortality on NELA v/s actual mortality is an important figure to compare to identify areas needing a particular focus. The number of patients turned down for reversal may well reflect the poor general health of the cohort and may have a relation to the low socioeconomic status of the population in this area. As expected, there is a significant correlation between age and ASA grade in relation to reversal rate. Our high comorbidity rate and high ASA grade may have contributed to the high leakage rate.

## Acknowledgments

### Conflict of interest

The authors declare that there is no conflict of interest.

### Ethical approval

The study was approved by the Audit Committee of the Lewisham and Greenwich Trust (approval ID: 6296).

### Consent to participate

Written informed consent was obtained from the participants in the study.

### Authorship

MF contributed to data collection and writing the article. PS, RK, and OO designed the methodology and PN reviewed the article.

## References

[1] Hartmann H Nouveau procédé d’ablation des cancers de la partie terminale du colon pelvien.. Trentieme Congres De Chirurgie; Strasburg, 1921.

[2] Garber A, Hyman N, Osler T (2014). Complications of Hartmann takedown in a decade of preferred primary anastomosis.. Am J Surg.

[3] Barbieux J, Plumereau F, Hamy A (2016). Current indications for the Hartmann procedure.. J Visc Surg.

[4] Vermeulen J, Coene PP, Van Hou NM, van der Harst E, Gosselink MP, Mannaerts GH, Weidema WF, Lange JF (2009). Restoration of bowel continuity after surgery for acute perforated diverticulitis: should Hartmann’s procedure be considered a one-stage procedure?. Colorectal Dis..

[5] Reinhart T Grundmann (2013). Primary Colon Resection or Hartmann’s Procedure in Malignant LeftSided Large Bowel Obstruction? The Use of Stents as a Bridge to Surgery.. World Journal of Gastrointestinal Surgery 5.1, Jan.

[6] Fitz-Henry J (2011). The ASA classification and peri-operative risk.. Ann R Coll Surg Engl.

[7] Daabiss M (2011). American Society of Anaesthesiologists physical status classification.. Indian J Anaesth.

[8] Albarran SA, Simoens Ch, Van De, Winkel N, da Costa PM, Thill V (2009). Restoration of digestive continuity after Hartmann’s procedure: ASA score is a predictive factor for risk of postoperative complications.. Acta Chir Belg.

[9] Maggard MA, Zingmond D, O’Connell JB, Ko CY (2004). What proportion of patients with an ostomy (for diverticulitis) get reversed?. Am Surg..

[10] van de Wall BJ, Draaisma WA, Schouten ES, Broeders IA, Consten EC (2010). Conventional and laparoscopic reversal of the Hartmann procedure: a review of literature.. J Gastrointest Surg.

[11] Melkonian E, Heine C, Contreras D (2017). Reversal of the Hartmann’s procedure: A comparative study of laparoscopic versus open surgery.. J Minim Access Surg.

[12] Keck JO, Collopy BT, Ryan PJ, Fink R, Mackay JR, Woods RJ (1994). Reversal of Hartmann’s procedure: effect of timing and technique on ease and safety.. Dis Colon Rectum.

[13] Constantinides VA, Heriot A, Remzi F, Darzi A, Senapati A, Fazio VW, Tekkis PP (2007). Operative strategies for diverticular peritonitis: a decision analysis between primary resection and anastomosis versus Hartmann’s procedures.. Ann Surg.

[14] Roig JV, Salvador A, Frasson M, García-Mayor L, Espinosa J, Roselló V, Hernandis J, Ruiz-Carmona MD, Uribe N, García-Calvo R, Bernal JC, García-Armengol J, García-Granero E; en representación del Grupo Cooperativo de la Sociedad Valenciana de Cirugía (2018). Stoma reversal after surgery for complicated acute diverticulitis: A multicentre retrospective study.. Cir Esp (Engl Ed).

[15] Horesh N, Lessing Y, Rudnicki Y, Kent I, Kammar H (2020). Timing of colostomy reversal following Hartmann’s procedure for perforated diverticulitis.. J Visc Surg.

[16] Royo-Aznar A, Moro-Valdezate D, Martín-Arévalo J, Pla-Martí V, García-Botello S (2018). Reversal of Hartmann’s procedure: a single-centre experience of 533 consecutive cases.. Colorectal Dis.

[17] Keck JO, Collopy BT, Ryan PJ, Fink R, Mackay JR, Woods RJ (1994). Reversal of Hartmann’s procedure: effect of timing and technique on ease and safety.. Dis Colon Rectum.

[18] Pearce NW, Scott SD, Karran SJ (1992). Timing and method of reversal of Hartmann’s procedure.. Br J Surg.

[19] Hallam S, Mothe BS, Tirumulaju RMR (2018). Hartmann’s procedure, reversal and rate of stoma-free survival.. Ann R Coll Surg Engl.

[20] Christou N, Rivaille T, Maulat C (2020). Identification of risk factors for morbidity and mortality after Hartmann’s reversal surgery - a retrospective study from two French centers.. Scientific Reports.

[21] Zarnescu EC, Zarnescu NO, Costea R, Rahau L, Neagu S (2015). Morbidity after reversal of Hartmann operation: retrospective analysis of 56 patients.. Journal of Medicine and Life.

[22] Siddiqui MR, Sajid MS, Baig MK (2010). Open *vs.* laparoscopic approach for reversal of Hartmann’s procedure: a systematic review.. Colorectal Dis.

